# Identification of patient-derived glioblastoma stem cell (GSC) lines with the alternative lengthening of telomeres phenotype

**DOI:** 10.1186/s40478-019-0732-4

**Published:** 2019-05-16

**Authors:** Ahsan Farooqi, Jie Yang, Vladislav Sharin, Ravesanker Ezhilarasan, Carla Danussi, Christian Alvarez, Sharvari Dharmaiah, David Irvin, Jason Huse, Erik P. Sulman

**Affiliations:** 1 University of Texas MD Anderson Cancer Center, Houston, TX USA; 2 NYU Langone Health, New York, NY USA

Glioblastoma multiforme (GBM) is an aggressive brain tumor with a poor overall prognosis. Current standard of care involves surgical resection followed by adjuvant treatment with radiation (RT), temozolomide, and tumor treating fields (TTF) [13]. Despite this aggressive treatment modality, median overall survival is approximately 15 months. Telomeres are terminal DNA elements found at eukaryotic chromosomal ends consisting of hexagonal repeats of (TTAGGG)_n_ which are essential for maintaining genomic stability [1]. To maintain telomere length and circumvent the end-replication problem, most cancer cells express telomerase [8]. Telomerase is composed of two subunits: a catalytic component with reverse-transcriptase activity encoded by the gene *TERT*, and an 11 base-pair RNA template encoded by the gene *TERC* [11]. Mutations in the promoter region for *TERT* occur in approximately 60–80% of GBM, leading to increased telomerase activity and enabling replicative immortality [10]. A defining feature of anaplastic astrocytomas and a small fraction of secondary GBM, is activation of a telomerase-independent alternative lengthening of telomeres (ALT) mechanism, driven by homologous recombination (HR) machinery [7]. ALT tumors can readily be detected by assaying for the presence of extrachromosomal telomeric DNA C-Circles (CCs) via qPCR or ALT-associated telomere foci by FISH on pathological specimens [6]. ALT+ high grade glioma (HGG) are enriched in tumors with loss of function mutations in *ATRX* (alpha-thalassemia/mental retardation X-linked) and less commonly, *SMARCAL1*. When these chromatin remodeling genes are inactivated, the resultant replication stress and aberrant HR at telomeres is hypothesized to lead to ALT [2]. Mutations in both *ATRX* and *SMARCAL1* are mutually exclusive with *TERT* promoter mutations suggesting functional redundancy between these two mechanistic pathways [3, 4].

Here, we sought to identify and characterize ALT+ GBM by screening through a panel of 24 patient-derived GBM stem cell lines (GSCs). We tested for ALT using a novel qPCR method that measures both telomere content (TC), which is indicative of overall telomere length, and DNA C-Circles (CCs), which are specific and quantifiable markers for ALT activity [9]. Telomerase expression was assessed by quantifying mRNA levels of *TERT* using whole transcriptome sequencing. ATRX protein expression was measured by immunoblotting.

Of the 24 GSCs that were tested, 2 were found to be ALT+ (8.3%), GS 5–22 and GS 8–18. These 2 cell lines have significantly elevated DNA CC content (*P* < 0.001, t-test) and telomere content (*p* < 0.001, t-test) relative to other GSCs (Fig. 1a and b). Furthermore, both GS 5–22 and GS 8–18 lack detectable full length ATRX protein upon immunoblot analysis (Fig. 1c). Whole transcriptome sequencing data (available for 22 of 24 GSCs) identified mRNA expression of *TERT* to be negligible in the two ALT+ GSCs, indicating absence of telomerase activity, whereas the remaining GSCs all had some quantifiable level of *TERT* expression (*p* = 0.0087, Mann-Whitney test) (Fig. 1d). Importantly, both GS 5–22 and GS 8–18 were derived from patients with secondary glioblastoma with concurrent *IDH* mutations. Also, p53 immunostaining was positive in both ALT+ GSCs (data not shown) corroborating p53 loss of function and mutant *IDH* along with ATRX loss as important in the development of ALT+ GBM. GS 5–22 and 8–18 display longer doubling times in vitro, 5 days and 8 days, respectively, relative to ATRX-intact *TERT*-positive GSCs which have a mean doubling time of ~ 3–4 days. We injected GS 522 cells intracranially into athymic mice to evaluate their ability to generate stable xenografts, and saw tumors form within 1 months’ time (Fig. 1e).

**Fig. 1:**
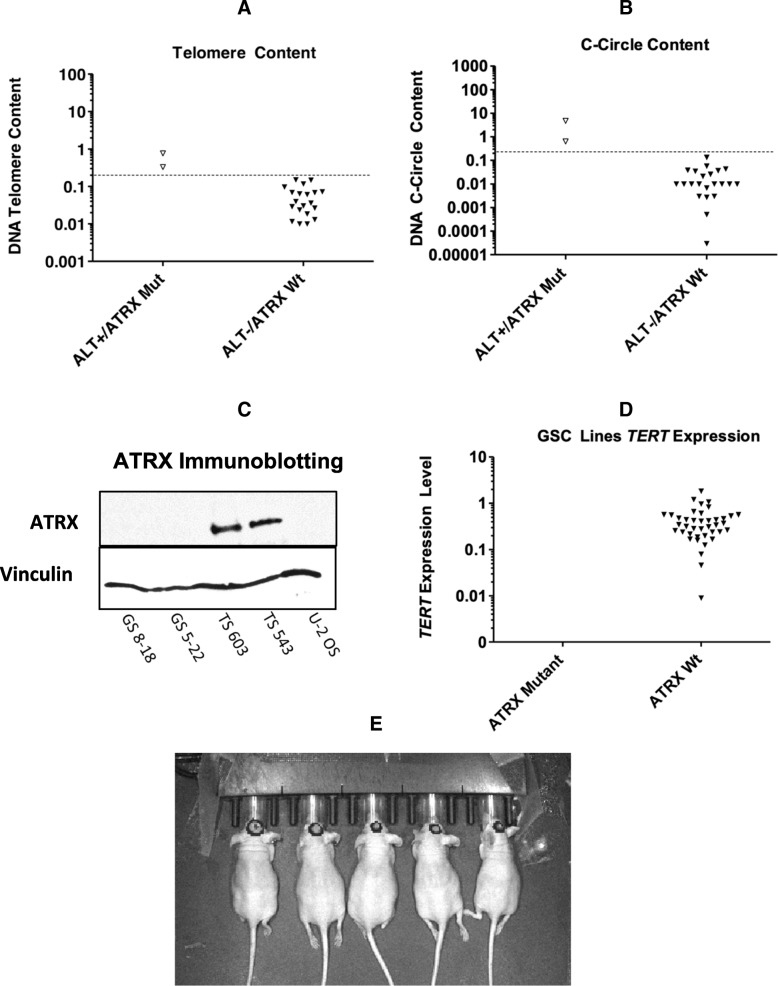
ALT+ GSCs were detected by quantifying telomere (**a**) and DNA C-Circle content (**b**) in a panel of 24 cell lines. Using a threshold cut-off value of 0.5 (dashed line) for telomere content and CCs, 2 ALT+ GSCs were identified, GS 8–18 and GS 5–22. Both GS 5–22 and GS 8–18 lack detectable ATRX protein (**c**). Additionally, these cell lines have negligible mRNA expression for *TERT* (**d**), indicating lack of telomerase activity. U-2 OS, a commercially available ALT+ osteosarcoma cell line which is *ATRX* mutant was used as a positive control for ALT and negative control for ATRX immunoblotting. Conversely, TS 603 and TS 543 which are known *ATRX* wild-type GSCs, were used as negative controls for ALT and positive controls for ATRX immunoblotting. GS 5–22 cells, stably expressing the luciferase reporter, were injected intracranially into nude mice and formed tumors within 4 weeks (**e**)

To date, only 2 ALT+ glioma cell lines have been documented (TG-20 and JHH-GBM14) [5, 12], however in these prior studies ALT was assayed for by immunofluorescent detection of telomere/PML body foci and lack of telomerase activity via the telomerase repeat amplification protocol (TRAP) assay. We report here that detection of DNA CCs via qPCR and mRNA quantification of *TERT* are also usable biomarkers that can reliably detect ALT and may be more applicable in a clinical setting as both assays require minute amounts of DNA and RNA. In conclusion, identification of these ALT+ GSCs will enable future explorations of the mechanisms and biology of the ALT phenotype, and will serve as pre-clinical models to test novel chemotherapeutic agents in an effort to improve outcomes in a subset of high-grade gliomas and secondary GBM.

## References

[1] Blackburn EH (1991). Structure and function of telomeres. Nature.

[2] Brosnan-Cashman JA, Graham MK, Heaphy CM (2018). Genetic alterations associated with ALTered telomeres. Oncotarget.

[3] Diplas BH, He X, Brosnan-Cashman JA, Liu H, Chen LH, Wang Z, Moure CJ, Killela PJ, Loriaux DB, Lipp ES, Greer PK, Yang R, Rizzo AJ, Rodriguez FJ, Friedman AH, Friedman HS, Wang S, He Y, McLendon RE, Bigner DD, Jiao Y, Waitkus MS, Meeker AK, Yan H (2018). The genomic landscape of TERT promoter wildtype-IDH wildtype glioblastoma. Nat Commun.

[4] Eckel-Passow JE, Lachance DH, Molinaro AM, Walsh KM, Decker PA, Sicotte H, Pekmezci M, Rice T, Kosel ML, Smirnov IV, Sarkar G, Caron AA, Kollmeyer TM, Praska CE, Chada AR, Halder C, Hansen HM, McCoy LS, Bracci PM, Marshall R, Zheng S, Reis GF, Pico AR, O'Neill BP, Buckner JC, Giannini C, Huse JT, Perry A, Tihan T, Berger MS, Chang SM, Prados MD, Wiemels J, Wiencke JK, Wrensch MR, Jenkins RB (2015). Glioma groups based on 1p/19q, IDH, and TERT promoter mutations in tumors. N Engl J Med.

[5] Heaphy CM, Schreck KC, Raabe E, Mao XG, An P, Chu Q, Poh W, Jiao Y, Rodriguez FJ, Odia Y, Meeker AK, Eberhart CG (2013). A glioblastoma neurosphere line with alternative lengthening of telomeres. Acta Neuropathol.

[6] Henson JD, Cao Y, Huschtscha LI, Chang AC, Au AY, Pickett HA, Reddel RR (2009). DNA C-circles are specific and quantifiable markers of alternative-lengthening-of-telomeres activity. Nat Biotechnol.

[7] Henson JD, Neumann AA, Yeager TR, Reddel RR (2002). Alternative lengthening of telomeres in mammalian cells. Oncogene.

[8] Kim NW, Piatyszek MA, Prowse KR, Harley CB, West MD, Ho PLC, Coviello GM, Wright WE, Weinrich SL, Shay JW (1994). Specific Association of Human Telomerase Activity with Immortal Cells and Cancer. Science.

[9] Lau LM, Dagg RA, Henson JD, Au AY, Royds JA, Reddel RR (2013). Detection of alternative lengthening of telomeres by telomere quantitative PCR. Nucleic Acids Res.

[10] Nonoguchi N, Ohta T, Oh JE, Kim YH, Kleihues P, Ohgaki H (2013). TERT promoter mutations in primary and secondary glioblastomas. Acta Neuropathol.

[11] Nugent CI, Lundblad V (1998). The telomerase reverse transcriptase: components and regulation. Genes Dev.

[12] Silvestre DC, Pineda JR, Hoffschir F, Studler JM, Mouthon MA, Pflumio F, Junier MP, Chneiweiss H, Boussin FD (2011). Alternative lengthening of telomeres in human glioma stem cells. Stem Cells.

[13] Stupp R, Taillibert S, Kanner AA, Kesari S, Steinberg DM, Toms SA, Taylor LP, Lieberman F, Silvani A, Fink KL, Barnett GH, Zhu JJ, Henson JW, Engelhard HH, Chen TC, Tran DD, Sroubek J, Tran ND, Hottinger AF, Landolfi J, Desai R, Caroli M, Kew Y, Honnorat J, Idbaih A, Kirson ED, Weinberg U, Palti Y, Hegi ME, Ram Z (2015). Maintenance therapy with tumor-treating fields plus Temozolomide vs Temozolomide alone for glioblastoma: a randomized clinical trial. JAMA.

